# Advances in the beneficial endophytic fungi for the growth and health of woody plants

**DOI:** 10.48130/forres-0024-0025

**Published:** 2024-08-20

**Authors:** Liang Hong, Qingao Wang, Junhao Zhang, Xuan Chen, Yuxin Liu, Fred O. Asiegbu, Pengfei Wu, Xiangqing Ma, Kai Wang

**Affiliations:** 1 College of Forestry, Fujian Agriculture and Forestry University, Fuzhou 350002, China; 2 Chinese Fir Engineering Research Center of National Forestry and Grassland Administration, Fuzhou 350002, China; 3 Department of Forest Sciences, PO Box 27, University of Helsinki, FIN-00014 Helsinki, Finland

**Keywords:** Endophytic fungi, Woody plant, Plant growth promotion, Stress tolerance, Dark septate endophytes (DSE)

## Abstract

In recent years, the importance of microorganisms for plant survival has been increasingly recognized. Endophytic fungi, as part of holobiont, can confer growth advantages to plants. Most studies have shown that the endophytic fungi of forest trees can promote host plant growth, increase adversity resistance, and thus improve the survival competitiveness of forest trees. However, the beneficial examples of endophytic fungi on the growth and development of woody plants have not been systematically summarized. This review is focused on various aspects of beneficial endophytic fungi in forest trees (definition, classification, colonization mechanisms, etc.), with an emphasis on their beneficial roles in woody plant growth, protection against biotic and abiotic stresses, as well as the response of forest trees to endophytic fungi. In addition, this review lists a series of experiments on screening beneficial endophytic fungi from Chinese fir (*Cunninghamia lanceolata*) and verifying their beneficial functions, to explore the mutualistic relationships between them. This review not only provides a theoretical basis for the study of beneficial endophytic fungi in forest trees in the future but also sheds light on the molecular perspectives for a mechanistic understanding of their potential future significance for the sustainable utilization of forest resources and ecological environment protection.

## Introduction

The forest is a complex community dominated by woody plants, which plays an important role in maintaining ecological balance, climate stability as well as biodiversity. Forests provide a large number of ecological niches for the growth and reproduction of various microorganisms (e.g. bacteria, fungi, protozoa) and nematodes. Accordingly, they can form complex symbiotic relationships with plants and contribute to their productivity and adaptability in the natural environment^[1]^. Plants have diverse and well-structured microbial communities that colonize almost every available plant tissue, including the rhizosphere (the root-soil interface of plants), the phyllosphere (the air-plant interface), and the endosphere (the internal tissues of plants)^[2]^. These microbial communities that colonize both inside and outside of plant tissues are collectively referred to as the plant microbiota (microbiome)^[3]^. Many studies have been conducted to unravel the importance of microorganisms in plant growth, and health^[4,5]^. The plant microbiome confers many adaptive advantages to the host such as growth promotion, nutrient uptake, stress tolerance, and resistance to pathogens. Similar to human and gut microbiota^[6]^, plants can be regarded as a holobiont or meta-organisms containing the host plants and their microbiota^[7,8]^. In addition, at the macro level, the plant microbiome has great potential for sustainable agriculture and for mitigating the negative effects of climate change on plant productivity^[9,10]^.

The plant microbiome comprises epiphytes as well as endophytes. The term 'endophyte' is a combination word derived from the Greek word 'endon' and 'phyton', the former meaning 'internal' and the latter meaning 'plant'. Initially, the German botanist Heinrich Friedrich Link was the first to use the term 'Entophytae' to describe endophytes in 1809 to denote a distinct group of parasitic fungi that live partially in plants. Later in 1886, the German botanist Anton de Bary, regarded as the father of plant pathology coined the term 'Endophyte' to describe microorganisms that colonize the internal tissues of stems and leaves^[11]^. These early definitions have undergone a long process of modification, for example, it has been clearly stated that not only stems and leaves of plants are colonized, but also roots and shoots of the host plants^[12,13]^. Meanwhile, the definition of endophyte was continuously revised by different researchers based on their understanding, and Hallmann et al.^[14]^ considered endophyte to be microbes that can be isolated from surface-sterilized plant tissues or extracted from the interior of plants and that do not visibly them. After this an endophyte was described as a microorganism that invades living plant tissues without causing any noticeable effect. It has also been argued that the term 'endophyte' should be considered in terms of habitat rather than function, and that the term should therefore be defined more broadly^[15]^. In other words, this means that endophytes may not remain endophytic throughout their life cycle. As representative members of the plant microbiome colonizing plant tissues, plant endophytes are more closely interdependent with their hosts due to their ability to interact directly with plant cells, and therefore they play a unique role in holobiont. Some endophytes are equally capable of improving soil fertility, promoting plant growth and development, and protecting plants from pathogens. In addition, some beneficial endophytes can induce tolerance mechanisms in hosts under biotic and abiotic environmental stresses through interactions with the host^[16,17]^.

As a member of the endophyte family, the mechanism by which endophytic fungi promote plant growth and development is related to the active substances they produce. These active substances may be able to inhibit the activity of pathogens that cause disease in the host, in a way that is analogous to the role of phytohormones in promoting the growth of the host to some extent, which will be elaborated on in more detail later. For example, an endophytic fungal strain *Trichoderma asperellum* LN004 can produce emodin interfering with the quorum sensing of *Pectobacterium carotovorum* subsp. *carotovorum* and reduces its pathogenicity, mitigating crop damage^[18]^. Similarly, dark septate endophytes promote the growth of host plants by producing growth hormones and volatile organic compounds that increase the biomass of plant rhizomes^[19]^. Researchers have continued to explore the molecular mechanisms involved in the growth, development, and induction of systemic resistance in woody plants by endophytic fungi based on previous studies^[20]^. However, not much is known about the biochemical, molecular, and genetic basis of endophytic interactions. One of the key ways to improve the utilization of woody plants is to deeply explore the mechanisms by which endophytic fungi regulate the growth, development, and resistance of woody plants.

This review therefore summarizes the recent advances on studies of endophytic fungi with respect to woody plants. We focus on the following three aspects: 1) basic understanding of endophytic fungi; 2) natural substances produced by plant endophytic fungi; 3) positive responses and practical applications of beneficial endophytic fungi on forest trees. It also describes the beneficial effects of endophytic fungi on woody plants from several perspectives, to provide a scientific basis for the use and conservation of woody plant resources.

## What are endophytic fungi?

### Extensive range of endophytic fungi

According to phylogenetic affiliation, metabolic potential, physiological characteristics, and mode of transmission, endophytes can be classified into systemic and non-systemic endophytes^[21]^. The term 'endophytic fungi' refers to fungi that live inside plant tissues by establishing a mutually beneficial symbiotic relationship with the host plants throughout all or part of their life cycle, without causing any adverse effects or diseases to the host^[22,23]^. Currently, endophytic fungi are present in all studied plants, 87% of endophytic fungi belong to *Ascomycota* and are distributed in 10 phyla, while the other 13% belong to phyla such as *Basidiomycota*, *Chytridiomycota*, *Mucoromycota* and *Zygomycota*^[24]^. Therefore, endophytic fungi form highly diverse and multifunctional microbial communities, which seem to be ubiquitous in nature. They have been isolated and cultured from underground and aboveground parts of a wide variety of plants. It is therefore not surprising that the biodiversity of endophytic fungi in both lower and higher plants exhibit variation across geographic regions and hosts^[25]^. Since the beginning of the 20^th^ century, with the deepening of research on endophytic fungi of different plant species, more and more endophytic fungi have been found in many broad leaf and conifer trees, which fully demonstrates the universality and diversity of endophytic fungi in forest trees further underlying its importance to the health of woody plants.

### Relationships between endophytic fungi and plants

In general, microorganisms associate with plants through three basic nutritional lifestyle strategies: Symbiosis, mutualism, and parasitism. Plant-endophytic fungi are often known to be symbiotic or mutualistic, although the presence of pathogenic has also been reported^[26]^. Mutualistic symbiosis is a positive and collaborative relationship in which both parties benefit. The endophytic fungi provide various benefits to host plants (such as promoting the acquisition of nutrients, promoting individual growth, and improving resistance to stress environment), and plants in turn provide carbohydrates to fungi. Carroll proposed two types of plant-endophyte mutualistic symbioses^[27]^: 'constitutive mutualism' and 'inducible mutualism'. As the name suggests, constitutive mutualism is a type of association exclusively between plants and microorganisms. This type of microorganism is usually found in herbs and can be passed on to the next generation through seeds, which is the case of the previously mentioned systemic endophytes. In contrast, if the endophytes exhibit inducible mutualistic symbiosis, then this usually refers to infecting the nutrient organs of the host (not involving transmission *via* host seeds). These non-systemic endophytes usually live in aging or metabolically inactive tissues, and only when the host is injured or affected by stress will they colonize important tissues.

Most plant-endophytic fungi interactions are considered to be symbiotic^[15,28]^. This type of combination has no known effect on the host plants, and the research progress at this stage is still uncertain whether it has an effect, although the endophytic fungi benefit from an uninterrupted nutrient supply. Many of the mutually beneficial symbionts and potential pathogens that can be induced during metabolically inactive phases may represent symbiotic states in plants. Some pathogenic microorganisms are known to spend part of their life cycle asymptomatically in a latent state within plant tissues^[29]^. Based on the definition of habitat endophytes^[14]^ (found within plant tissues without any visually visible symptoms of disease), we consider the potential pathogenic state of such endophytic fungi to be endophytic as well. However, microorganisms present as endophytes in a given plant may exhibit pathogenic symptoms in the same or similar plants. Endophytic fungi present in a specific host are usually similar at the phylogenetic level to harmful pathogens in related host species^[27]^. It has been reported that *Colletotrichum tofieldiae* inhibits the growth of *Capsella rubella* as a pathogen, whereas the former can act as a beneficial fungus to promote the growth of *Arabidopsis thaliana* under low phosphorus conditions^[30]^. This suggests that endophytic fungi can remain beneficial or turn into pathogens, depending on different environmental conditions or host plant types. In other words, microorganisms pathogenic to this plant species may exist as endophytic fungi in other species. However, plant-endophytic fungal symbioses may transform into mutually beneficial or pathogenic interactions depending on different external conditions. That is, although many studies have found some endophytic fungal isolates to be beneficial to host plants, it is important to note that endophytic fungi may also be pathogenic or potentially pathogenic, depending on environmental factors and host susceptibility.

### Colonization process of endophytic fungi

**Figure 1:**
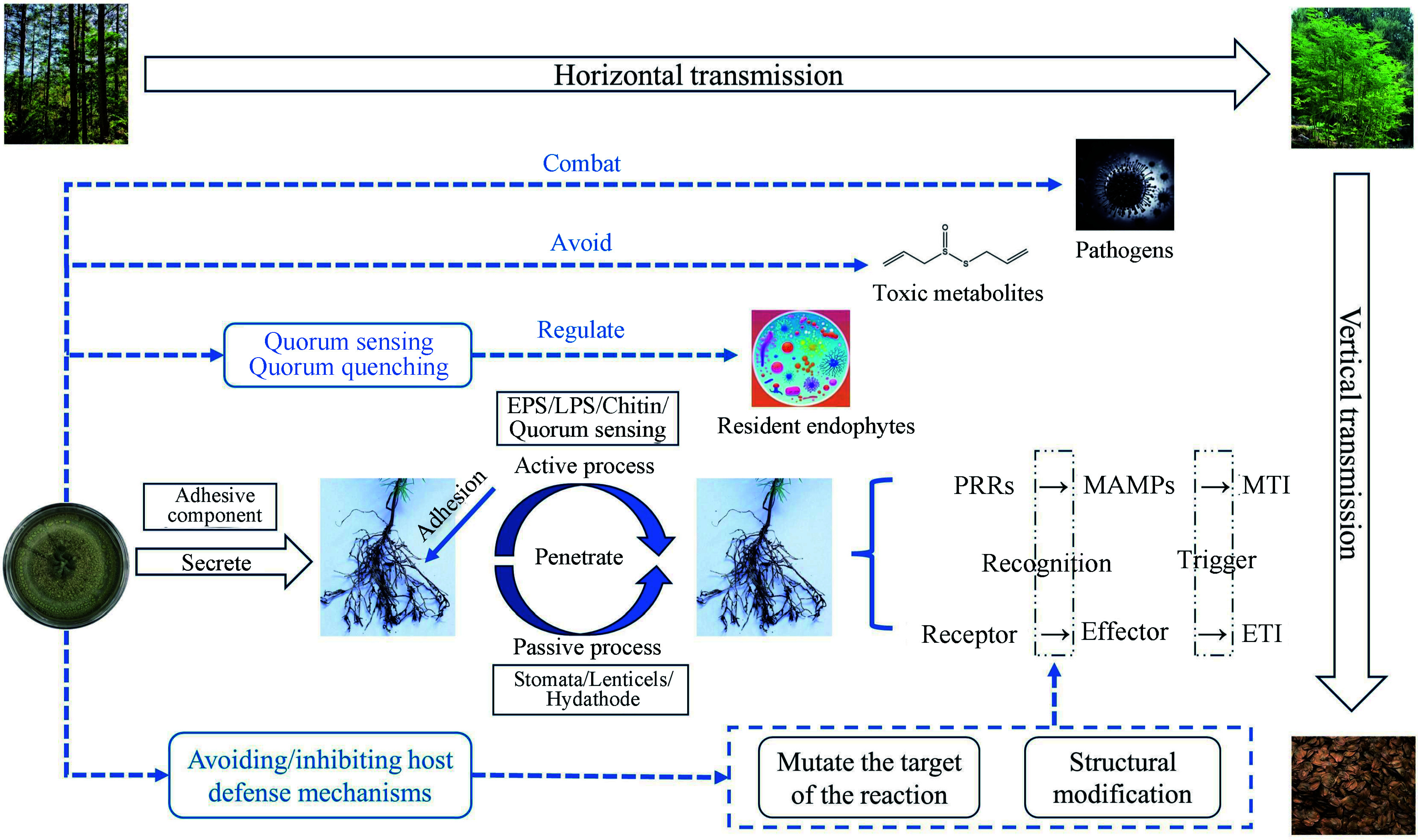
Colonization process and strategy of endophytic fungus transmission. EPS: Extracellular polysaccharides, LPS: Lipopolysaccharides, PRRs: Pattern recognition receptors, MAMPs: Microbe-associated molecular patterns; MTI: MAMP-triggered immunity; ETI: Effector-triggered immunity.

Most endophytic fungi originate from the host plant's growing environment, including soil and rhizosphere microorganisms, fungal spores floating in the air, and the feeding behavior of insects and animals^[31]^. Endophytic fungi usually enter plants *via* two modes of transmission (Fig. 1). The first is a vertical transmission, which is the main way of infecting offspring^[32]^. When seeds germinate under appropriate conditions, endophytic fungi from the parent enter the offspring plant during germination, thus enabling the spread of endophytic fungi between the host plants and the offspring. The second is a horizontal transmission, in which some endophytic fungi in the host plant horizontal transmission among different individuals through other propagators such as spores and/or hyphae, to realize the diffusion of endophytic fungi among plants.

Attachment or adhesion of fungal propagules is a prerequisite for the successful colonization of plants by endophytic fungi, and endophytic fungi follow a similar pattern of adhesion and penetration of host tissues as pathogenic microorganisms. Each endophytic fungus has a different colonization method and 'access'. Endophytic fungi actively exude adhesion components that help to recognize and bind to specific host plants in specific environments. Electron microscope study on the spores of endophytic fungus *Discula umbrinella* colonized in leaves showed that there was a sticky sheath composed of polysaccharide on the surface of its conidia, which could make endophytic fungi attach to host plants^[33]^. Shortly after the microbial strain has landed, plants then show different responses, which will lead to a diverse gene expression. In the initial stage, after the endophytic fungi are attached to the root surface, penetration structures are developed to facilitate colonization of the internal tissues. Penetration of plant surface could be through active or passive processes. Passive pathways include root fissures, stomata, lenticels, drains, or shoot incisions^[34]^, whereas active pathways are mediated by extracellular polysaccharides (EPS), lipopolysaccharides (LPS), structural components (e.g. chitin), and quorum sensing^[35]^. It has been reported that microorganisms select thin surface areas (including root hairs or the elongation zone of the root apical meristem (RAM)) to enter the root and produce a variety of extracellular hydrolytic enzymes (cellulases, hemicelluloses, pectinases, etc.) to directly penetrate the cell wall^[36]^.

Although in most cases plant-endophytic fungi interactions are beneficial to the host itself, plants still detect signals from microbes and respond appropriately by activating the plant immune system. Plants rely on innate immunity to recognize microbial signaling molecules, which leads to two distinct defense systems: 1) Plants recognize microbe-associated molecular patterns (MAMPs) *via* pattern recognition receptors (PRRs) localized on the cell surface, which leads to MAMP-triggered immunity (MTI); and 2) intracellular receptors in plants recognize molecules produced by microorganisms (known as effectors), which activate effector-triggered immunity (ETI) response^[37]^. It is easy to understand that beneficial microorganisms may temporarily circumvent the plant's self-protection mechanisms by evading ETI, or evade recognition by removing or mutating the recognized effectors to facilitate successful colonization of the host root.

As mentioned above, for the MTI defense system, the response of plants to the very first infections by endophytic fungi is similar to the response of plants to the invasion of pathogenic fungi. In this system, endophytic fungal communities can adapt the behavior of their interactions with the host plants in a self-serving way, thereby maximizing their fitness^[3]^. Structural modification and mutating the target of the reaction are the most effective methods. A MAMP originating from the fungal cell wall, β-glucan, is capable of triggering the plant immune system. For example, the FGB1 gene of root endophyte *Piriformospora indica* encodes a secreted fungal specific β-glucan binding lectin, which may change the composition and characteristics of fungal cell walls, thus effectively inhibiting the immunity triggered by β-glucan from different host plants^[38]^. These findings suggest that endophytic fungi are inherently resistant to defenses generated by their host plants, providing us with a deeper understanding of host-microbe coevolution.

The non-pathogenicity of endophytic fungi in their hosts is related to the homeostasis of the microecological environment between the endophyte and the host. The mechanisms underlying the interactions between endophytic fungi and the plant immune system are cumbersome, and research in this area is currently limited. The current more complete evolutionary strategies of endophytic fungi from entry to successful colonization in the host can be summarized as either: 1) avoidance and/or suppression of host defense mechanisms and protection from other toxic metabolites produced by the host, or 2) regulation of the growth and virulence of the resident endophytes through quorum sensing and quorum quenching^[39]^ (Fig. 1). We still need to dig deeper into the colonization mechanisms of endophytic fungi within host plants, which will help to facilitate the understanding of the symbiotic relationship between endophytic fungi and host forest trees, with potential for practical application.

## Natural substances produced by endophytic fungi

As an important microbial resource, endophytes not only live in plant tissues without causing disease but also produce a large number of natural active compounds. It has been often misinterpreted that these compounds originate from plants^[40,41]^. Endophytic fungi are an important class of microorganisms and also one of the least studied. They enhance host resistance to abiotic stresses, diseases, insects, pathogens, and herbivores by producing secondary metabolites with a wide range of biological activities^[42]^. It is no exaggeration to say that endophytic fungi mark the academic discovery of new reservoirs of chemicals that play unique roles that cannot be replaced by other microorganisms.

**Figure 2:**
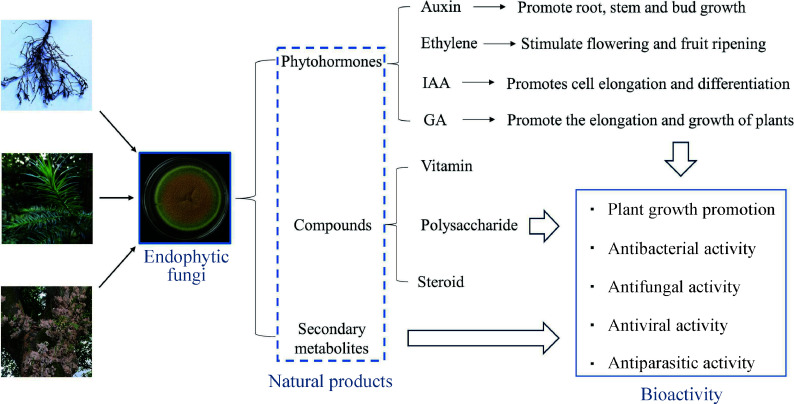
Natural substances produced by endophytic fungi. IAA: Indole acetic acid, GA: Gibberellins.

In recent years, the isolation of endophytic fungi and the study of their natural products have received much attention. Endophytic fungi represent a great potential for biodiversity, and they are considered to be a powerful source of new bioactive compounds^[43]^. Due to the interconnections between endophytic fungi and their hosts, many of the bioactive compounds produced by the former have been classified into different categories^[44]^, including, but not limited to, compounds such as vitamins, polysaccharides, and steroids^[45]^, phytohormones such as indole acetic acid (IAA), gibberellins (GA)^[46]^, and secondary metabolites^[47]^ (Fig. 2). As an example, IAA, a phytohormone with the chemical formula C_10_H_9_NO_2_, plays a pivotal role in coordinating a wide range of growth and developmental processes and physiological responses (e.g. cell elongation, division, and differentiation) in plants. Interestingly, with the intensive study of plant endophytic fungal products, it has been found that certain fungi also possess the ability to produce IAA. Corradi et al. found that the leaf-endophytic fungus *Pestalotiopsis* aff. *Neglecta*, isolated from Cupressaceae, produces IAA under *in vitro* conditions, and the production of IAA is significantly increased when co-cultured with specific bacterial strains^[48]^. It has been shown that *Aureobasidium* sp. BSS6 and *Preussia* sp. BSL10 isolated from foliage and stems of frankincense trees showed high potential for IAA production^[49]^. To verify this discovery, *Preussia* sp. BSL10 was applied to the host seedlings, and the stem length, leaf number, internodes, and photosynthetic pigment content of the young trees were found to be significantly increased.

These fungi can produce a large number of chemically diverse secondary metabolites that are known to have antimicrobial, antiparasitic, anticancer or antiviral activities^[50]^ (Fig. 2). Some endophytic fungi produce more than 12 metabolites similar to those produced by the host plants, including alkaloids, flavonoids, saponins, peptides, phenolic acids, terpenes, steroids, and other active compounds, and are considered to be potent sources of new compounds^[51]^. In addition, there are several reports suggesting that endophytic fungi produce substances originating from different biosynthetic pathways and belonging to different structural groups (e.g., terpenoids, steroids, xanthones, quinones, phenols, isocoumarins, tetralones, and cytochalasins)^[52]^. Among terpenoids, sesquiterpenes are a very diverse and widely distributed subclass of biologically important natural organic compounds in the plant kingdom. Tremulenolide A is a sesquiterpene first isolated from the liquid culture of aspen rotting fungus *Phellinus tremulae* in quaking aspen^[53]^, and it can also be isolated from the fermentation product of endophytic fungus *Flavodon flavus* PSU-MA201 from mangroves^[54]^. The isolation of tremulenolide A from mangrove endophytic fungal cultures was carried out in the same way as for *Pestalotiopsis* spp. PSU-MA92 and PSU-MA119^[55]^. Some researchers analyzed the antibacterial activity of tremulenolide A against *Staphylococcus aureus* ATCC25923 and *Cryptococcus neoformans* ATCC90113 using a colorimetric broth microdilution assay and found that this sesquiterpene exhibited antibacterial and antifungal activity with a minimum inhibitory concentration value of 128 μg/mL^[54]^. Most of the new natural compounds, except for the antibacterial activity of tremulenolide A, were found to be of great medicinal value.

The fact that the unique medicinal composition of plants allows endophytic fungi to produce higher-value natural products, has led to the recognition that they may have a better potential for application. In the past 20 years, many novel bioactive compounds with antimicrobial, antioxidant, and anticancer properties have been successfully isolated and characterized worldwide. This series of breakthroughs not only meets the growing needs of people but also has great significance for the conservation and regeneration of rare woody plant resources.

## Beneficial effects of endophytic fungi on forest trees

### Promotion of growth and development of woody plants by endophytic fungi

Phytohormones play an important regulatory role in almost all processes of plant growth and development. As mentioned in the previous section, plant endophytic fungi have the ability to produce specific phytohormones. Several reports have shown that the interaction of endophytic fungi with their host plants can produce a wide range of phytohormones that are used as exogenous substances to promote plant growth^[56]^. In addition to IAA, endophytic fungi of olive trees are known for their potential to promote plant growth^[57]^, which depends on improved nutrient supply and/or production of phytohormones, such as auxin in *Discosia* sp^[58]^.

Endophytic fungi also produce other substances that contribute to the growth of host plants, and although they are not classified as phytohormones, they still have a positive impact on plants that cannot be ignored. For example, common endophytic fungi *Lasiodiplodia* spp., isolated from the leaves of 32 different tree species, not only showed antifungal activity but also produced various types of enzymes such as cellulase and β-glucosidase enzyme^[59]^. This suggests that *Lasiodiplodia* spp. from different hosts both have potentially beneficial effects on different trees. Previous studies have shown that as a saprophytic fungus that degrades fallen leaves in dry deciduous forests, *Lasiodiplodia* can utilize phenylpropanoid precursors and salicylic acid to control host defense, suggesting that they may influence physiological processes in the host plant^[60]^. Inoculation with endophytic fungi *Trichoderma* sp. PDR1-7 significantly increased the availability of soil nutrients and growth of Scots pine seedlings, chlorophyll and protein content, and superoxide dismutase (SOD) activity, probably because *Trichoderma* sp. releases organic acids and siderophores^[61]^.

It is undeniable that the endophytic fungi themselves have properties that affect the growth of the host plants. It was shown that 15N content in cacao leaves inoculated with both the pathogenic *Mycosphaerella* spp. and the protective endophytic fungi *Colletotrichum tropicale* was significantly higher, and that mixed endophyte-pathogen infections also increased the total biomass. This finding is consistent with previous findings in the same cacao-*C. tropicale* system, which documented a variety of localized effects of *C. tropicale* on plant leaves, including altered expression of 115 nitrogen metabolism genes in cacao^[62]^. Isolates P06 belonging to Helotiales and P04 of *Serendipita vermifera* both increased the number of root tips and shoot biomass in poplar seedlings^[63]^. These endophytic fungi confer plants' resistance to heavy metals in the external environment, so they can be considered for use in phytoremediation technologies for heavy metal-contaminated soil.

Different endophytic fungi can also reach the expectation of promoting plant growth and development through synergistic or inhibitory effects with each other. Field inoculation with the arbuscular mycorrhizal fungi (AMF) and the endophytic fungi *Piriformospora indica* was able to partially improve the external (number of fruits per tree and fruit size) and internal (glucose, sucrose, and fructose) qualities of Newhall navel orange^[64]^. Moreover, branch elongation of young apple trees inoculated with AMF *Paraglomus* sp. SW1 was accompanied by an increase in the number of AMFs, a significant decrease in the number of *Fusarium* spp. in the soil, which resulted in an effective mitigation of the negative effects of apple replant disease^[65]^.

### Endophytic fungi assist woody plants to cope with biotic stress

Plants are inevitably subjected to stress and interference from various biological factors in the environment during their growth, which will result in poor plant growth or even death. Among these factors, biological stress includes biological interference such as pathogenic microorganisms or harmful insects^[66]^. Most studies have shown that endophytic fungi can not only promote plant growth but also improve plant resistance.

Certain endophytic fungi may increase the resistance of forest trees to biotic stresses caused by herbivores. The Siberian elm inoculated with endophyte isolate YCB36, an Ascomycete showed decreased proline content, increased stomatal conductance of leaves, and higher concentrations of flavonoids and total phenols in xylem tissues, which indicated that YCB36 induced defensive metabolism of host plants^[67]^. At present, it is known that some fungal infections are helpful in improving the tolerance of trees to herbivores such as pests. Compared with those without endophytic fungi, the survival rate of juvenile eastern spruce budworm and the amount of fallen needles of trees inoculated with *Phialocephala scopiformis* were decreased, which indicated that *P. scopififormis* could effectively control the budding insects of eastern spruce^[68,69]^. In addition, in the Acadian forests of Canada's coastal provinces, forest plants provide nutrients and shelter for coniferous foliar endophytic fungi with 'foraging ascomycetes', which increase host survival by promoting tolerance of host plants to herbivorous insects or needle pathogens^[70]^.

When it comes to diseases, endophytic fungi also have excellent biocontrol potential. First, they may compete with pathogens for ecological niches by producing toxic substances that inhibit the pathogenic effects of pathogens. For example, needles of white spruce and white pine infected by toxic endophytic fungi contain varying concentrations of secondary metabolites that are toxic to needle pathogens^[71]^. The endophytic fungal community of New Zealand apples has the potential to reduce infection by the pathogen *Neonectria ditissima*, which could potentially be developed as a biological control strategy for European canker^[72]^. The endophytic fungi *Lophodermium nitens*, which produces the antifungal compound pyrenophorol, may increase the tolerance of eastern white pine to white pine blister rust^[73]^. The endophytic fungi studied showed extensive ecological niche overlap with the pathogen, suggesting that some endophytic strains may protect elm trees from Dutch Elm Disease (DED)-pathogens by competing for substrates, which provides a new tool for biological control of DED^[74]^. Endophytic fungi may improve the growth of American beech by altering the direction of disease progression through feedback on the major causal agents or by changing the symptomatic expression of beech bark disease and rates of tree decline^[75]^. Secondly, endophytic fungi may directly promote host plant resistance to pathogens. In natural habitats, endophytic fungi are beneficial in helping Brazilian rubber trees to resist pathogenic fungi, and they may exhibit the same ecological functions as antagonists of fungal pathogens in the interleaf^[76]^. Exposing cocoa seedlings to the leaves of healthy adults enriched the microbiome of the seedlings with *Colletotrichum tropicale*^[77]^. This endophytic fungus enhanced the resistance of the seedlings to pathogens by up-regulating the host's defense pathway, and the results showed that the seedlings experienced less pathogen damage after exposure^[77]^. The experimental objects are also woody plant cacao tree and endophytic fungus *Colletotrichum tropicale*. Inoculation of endophyte-free cacao leaves with *C. tropicale*, the predominant leaf endophyte fungi in healthy cocoa, increasing lignin and cellulose content of the inoculated cocoa and a cocoa gene (Tc00g04254) highly up-regulated by *C. tropicale* conferred resistance of host tissues to pathogen damage^[78]^. Furthermore, the incidence and severity of damage in silver poplar pretreated with mixed cell suspensions of endophytic fungi (mainly Pleosporales, Dothideales, and Eurotiales) were lower than in the control group, proving that the former had a high level of tolerance to Blackstar^[79]^. It was suggested that the inoculated endophytic fungal community in silver poplar enhances the host's tolerance to the pathogen, which could be because certain endophytic fungi contribute to defenses downstream of plant genetic resistance mechanisms^[79]^.

### Endophytic fungi assist woody plants to cope with abiotic stress

Abiotic stresses are usually inanimate natural environmental factors, such as sunlight, temperature, or wind, which may cause harm to plants and animals in affected areas. Abiotic stress will affect animals, while plants are especially or even completely dependent on environmental factors, so abiotic stress has a particularly obvious restriction on plants. Plant microbiome can improve plant access to and supply of nutrients and alleviate abiotic stress^[80]^. It has been shown that endophytic fungi enhance plant adaptation to heavy metal stress by mechanisms including co-evolutionary strategies, immune modulation and detoxification translocation^[81]^.

**Figure 3:**
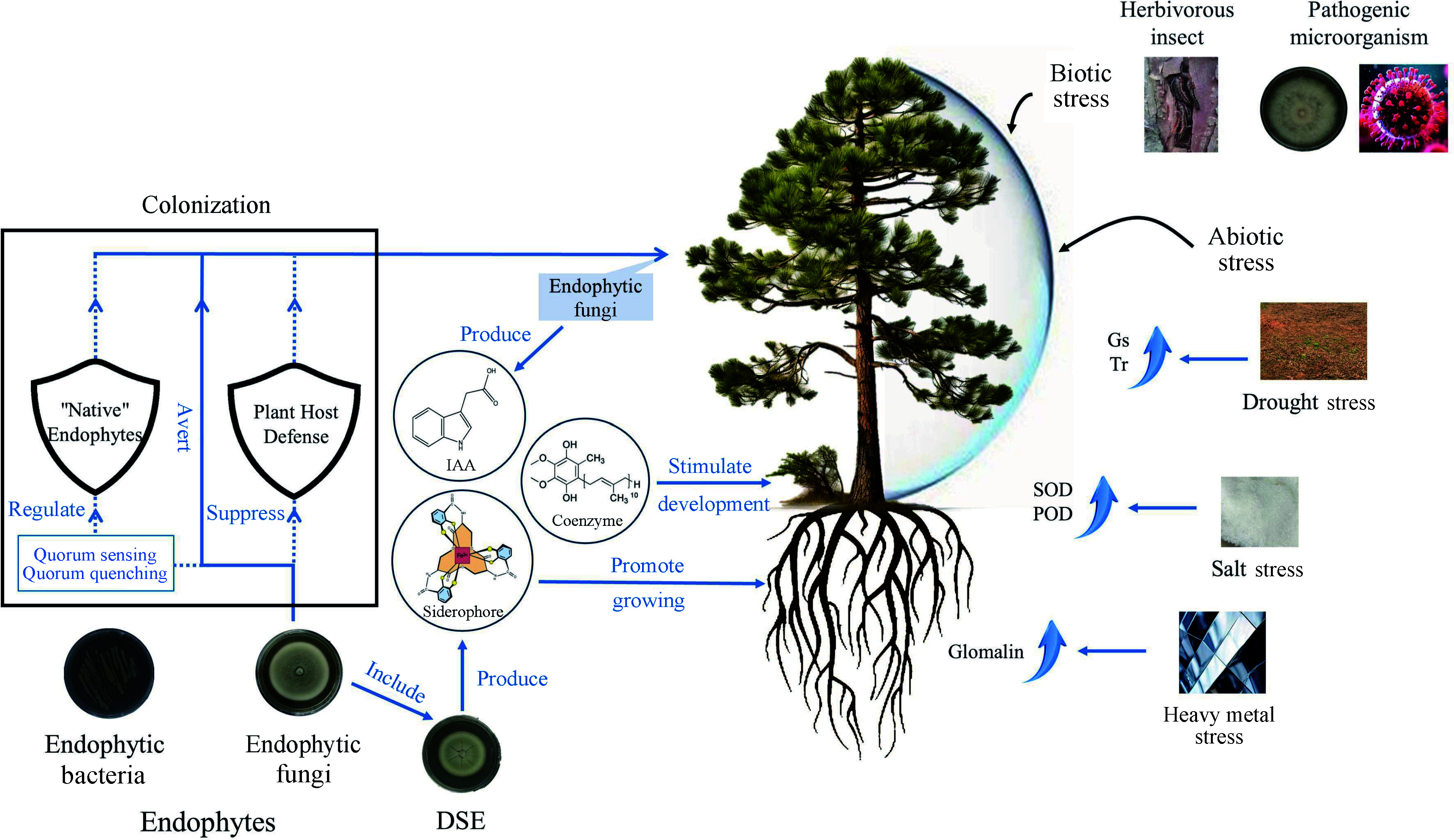
Ways to promote the growth and development of woody plants by endophytic fungi. Gs: Stomatal conductance, Tr: Transpiration rate, IAA: Indole acetic acid, SOD: Superoxide dismutase; POD: Peroxidase; DSE: Dark septate endophytes.

Several studies have shown that the presence of endophytic fungi is more favorable for better adaptation of host plants in salt-stressed environments. Inoculation with the endophytic ascomycete *Curvularia* sp. isolated from the saline plant *Suaeda salsa* resulted in a simultaneous increase in the activities of SOD and ascorbate peroxidase in Chinese white poplar, indicating that this endophytic fungus could alleviate the effects of salinity stress on white poplar^[82]^. It was also observed that the colonization of endophytic fungi belonging to the phylum Leotiomycetes and Basidiomycota was increased at sites with higher total phosphorus content. This observation confirms the ability of endophytic fungi, which are usually known to form ectomycorrhizal structures on the roots of European alder under salt stress conditions, to help the host to adapt to salt-stressed environments by accumulating proline and antioxidants and maintaining the plant's water potential^[83]^. It has been demonstrated for the first time that expression of the TaACCD gene from the bioprophylactic endophytic fungus *Trichoderma asperellum* ACCC30536 resulted in elevated levels of SOD and peroxidase (POD) and reduced reactive oxygen species accumulation and ethylene synthesis in transgenic poplars compared to non-transgenic plants, which may be responsible for the enhanced salt tolerance of the host^[84]^ (Fig. 3). In addition, endophytic fungi can assist the host to survive in other stressful environments. Experiments shown that root endophytic fungi (including *Phialocephala fortinii*, *Rhizodermea veluwensis*, and *Rhizoscyphus* sp.), in addition to supporting *Clethra barbinervis* to enhance growth and promote K^+^ uptake, also increases plant tolerance to heavy metals by secreting glomalin, which binds heavy metals to exclude them from the roots^[85]^ (Fig. 3). Without the root endophytic fungi, *C. barbinervis* has difficulty growing in heavy metal-contaminated environments and exhibits shrinkage, a symptom of heavy metal poisoning^[85]^*.*

### Beneficial effects of dark septate endophytes (DSE) on woody plants

There is a kind of endophytic fungi that should be mentioned separately, the dark septate endophytes, which are characterized by having black, septate hyphae, and all belong to the group of ascomycetes. The DSE colonize intra- or inter-cellularly in the root, and they are globally distributed, being present in much greater numbers in stressed environments^[86]^.

Although little is known about the number, classification, and physiological characteristics of this fungal group, it is important that DSE play multiple roles in regulating plant growth, signaling, and stress tolerance^[87]^. Like other endophytic fungi, DSE promotes the growth and development of woody plants. It has been shown that inoculation of *Hedysarum scoparium* with different DSE was able to either promote the height and shoot biomass or enhance the stem meristem and total biomass^[88]^. Secondly, DSE can act as biocontrol fungi to assist tree hosts against harmful pathogens. Infection of Norway spruce seedlings with the DSE *Phialocephala sphaeroides* and/or the root pathogen *Heterobasidion parviporum* revealed that DSE promoted root growth and counteracted the effects of *H. parviporum* on the growth of spruce seedlings^[89,90]^. The negative effect of *H. parviporum* on spruce seedling growth was attributed to the strong inhibitory effect of the presence of DSE on *H. parviporum* transcripts encoding genes related to nutrient acquisition^[89,90]^.

There are examples of the potential of DSE as beneficial endophytic fungi particularly in the context of abiotic stresses. It was shown that inoculation with both DSE (*Cladosporium* sp. and *Rhyzopycnis vagum*) not only increased the height of the physic nut in Pb-contaminated soil by 24.1% and chlorophyll content by 11% to 33%, but also resulted in a higher translocation factor value in the host^[91]^. Nine DSE strains isolated from the super arid shrub *Gymnocarpos przewalskii* and inoculated with sand holly, one *Darksidea* strain, *Knufa* sp., and *Leptosphaeria* sp. increased total sand holly biomass; two *Paraconiothyrium* strains, *Phialophora* sp. and *Embellisia chlamydospora* had a significant positive effect on the number of plant branches, potassium and calcium content^[92]^. This suggests that some DSE strains possess the ability to promote the growth of desert woody plants, indicating that they can be used for vegetation restoration in arid environments.

For drought-stressed environments, inoculation with DSE can help plants adapt to stress by altering root morphology, mitigating ultrastructural damage, and influencing the balance of endogenous hormones, which is important for the cultivation and preservation of red bean trees^[93]^. The pigment content, net photosynthetic rate, stomatal conductance (Gs) and transpiration rate (Tr) of inoculated seedlings were significantly higher than those of non-inoculated seedlings when inoculated with DSE *Acrocalymma vagum* under sterile drought stress^[94]^ (Fig. 3). It indicates that DSE plays a positive role in the growth and drought tolerance of the seedlings of red bean trees.

In summary, endophytic fungi interact closely with woody host plants, which provide nutrients and shelter to endophytic fungi, while endophytic fungi secrete bioactive substances to enhance plant growth promotion, increase seed production and strengthen the resistance of forest trees to biotic and abiotic stresses^[95,96]^. In general, how endophytic fungus contributes positively to woody plants can be broadly categorized as follows: Promoting the growth and development of the trees themselves, responding to biotic stresses and responding to abiotic stresses (Fig. 3).

### Studies related to endophytic fungi of *Cunninghamia lanceolata*

Our group has isolated and purified a variety of endophytic fungi from different parts of *C. lanceolata* (sapwood, fine roots, coarse roots, etc.) after multi-step surface disinfection. Among these, 22 species have been identified. By reviewing various literatures, we found *Talaromyces. verruculosus* and *T. yunnanensis*, *Penicillium glabrum*, *Trichoderma lixii*, *Cladosporium sphaerospermum*, *Chaetomium globosum* showed favorable characteristics in different host plants, such as promoting plant growth, solubilizing soil organic/inorganic phosphorus, inhibiting plant pathogens, and tolerating heavy metals or saline environments.

**Figure 4:**
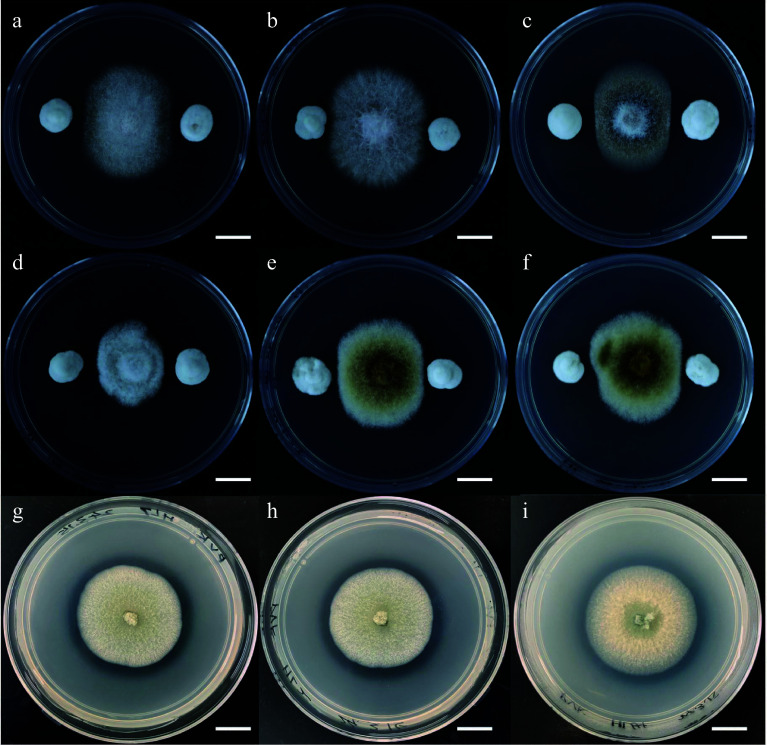
Experiments on the potential beneficial functions of endophytic fungi of *Cunninghamia lanceolata.* (a)−(f) Antagonism experiments of endophytic fungi against six fir pathogenic fungi, the pathogenic fungi are *Fusarium concentricum*, *Fusarium fujikuroi*, *Colletotrichum gloeosporioides*, *Colletotrichum cangyuanense*, *Alternaria kunyuensis*, and *Alternaria shangdongensis* in order. (g)−(i) Inoculation of *C. lanceolata* endophytic fungi on Pikovskaya medium to test their ability to solubilize inorganic phosphorus. Bar = 1 cm.

To search for all endophytic fungi isolated from *C. lanceolata* that may have beneficial functions, our group conducted a series of screening-related experiments. For example, we conducted antagonism experiments against pathogenic fungi in an *in vitro* medium environment with all the endophytic fungi (Fig. 4). Six known *C. lanceolata* pathogenic fungi were selected and inoculated in the center of Potato Dextrose Agar medium, and then other endophytic fungi isolated from different parts of *C. lanceolata* were inoculated into the four corners of the medium. The antagonistic effect of the endophytic fungus on the pathogen was determined by the distance between the pathogens and the endophytes and/or their growth trends after 4 d. Finally, we found that *P. glabrum* had a significant antagonistic effect on all six pathogens. In addition, our group conducted experiments on the dissolution of organic and inorganic phosphorus (Fig. 4). We also inoculated the endophytic fungus on Pikovskaya medium and Mongina medium respectively. We determined whether the endophytic fungi secreted some phosphorus-solubilizing substances that diffused to the surroundings by observing whether a transparent circle formed around the colony. Subsequently, the diameters of the colonies and the phosphorus solubilizing circles were measured to understand the phosphorus solubilizing ability of these endophytic fungi. We found that *P. glabrum* formed hyaline rings on both media, suggesting that it might have the ability to antagonize pathogenic fungi and dissolve phosphorus, which had positive effects on the growth and health of *C. lanceolata*.

Meanwhile, we have selected target endophytic fungal strains with potential beneficial functions for *C. lanceolata* growth promotion and *C. lanceolata*-pathogen-endophytic fungus interactions. Fir seedlings were used as test plants in sterile Petri dishes, greenhouse pots, and field plots, and were inoculated with the target endophytic fungus alone or co-inoculated with the target endophytic fungus and fir pathogens. Plant growth, root morphology, photosynthetic physiology and other indicators were measured after a specific time of planting. Physical morphology and photosynthetic properties as well as nutrient changes from seed to seedling of *C. lanceolata* were used to investigate the effects of endophytic fungi with potential beneficial functions (in the presence of pathogens) on fir hosts. Aiming at the problems of large-scale withering and succession cropping obstacles of *C. lanceolata* in some areas, we hope to find effective endophytic fungal strains with potential utilization advantages and provide a scientific basis for promoting the growth of *C. lanceolata* under natural conditions.

## Future perspectives

With the continuous progress of techniques, our research on beneficial endophytic fungi in woody plants will move to a new stage: 1) To carry out studies on the colonization and function of single endophytic fungus, we will gain a deeper understanding of the effect of host plant-specific screening on the colonization process of endophytic fungi; 2) To use microbiome engineering to select beneficial endophytic fungi as synthetic communities and use them in forest nurseries; 3) By studying the genetic characteristics of endophytic fungi in detail, we can target the modification of endophytic fungi with better plant growth-promoting properties through gene editing and other techniques; 4) From a host perspective, genetic techniques can also be used to select woody plants that are most likely to harbor beneficial endophytes. These directions can not only further improve the quality of forest trees, but also provide new possibilities for improving the efficiency of forestry production. The potential practical application of endophytic fungi in forestry has been widely envisioned. Endophytic fungi will provide trees survival advantages in terms of promoting the growth and development of forest trees as well as resisting biotic and abiotic stresses, including through the production of a variety of metabolites. This review provided new knowledge for scientific research related to the growth and development of woody plants. Future studies can further explore how different beneficial endophytic fungi can be utilized to promote the growth of forest trees and the health of forest ecosystems. We hope that the findings of this article can provide effective references for researchers and practitioners in related fields, and provide theoretical support and practical guidance for the conservation and utilization of forest resources.

## Author contributions

The authors confirm contribution to the paper as follows: study conception and design: Wang K, Hong L; literature collection: Hong L, Wang Q, Zhang J, Chen X, Liu Y; figure preparation: Hong L; draft manuscript preparation: Wang K, Hong L; manuscript revision: Asiegbu F, Wu P, Ma X, Wang K. All authors reviewed the results and approved the final version of the manuscript.

## Data availability

The datasets generated during and/or analyzed during the current study are available from the corresponding author on reasonable request.
